# Gaso-transmitters: expanding the kinetic universe of cell signaling

**DOI:** 10.1152/ajpcell.00323.2016

**Published:** 2017-01-01

**Authors:** Jeffrey S. Isenberg, Josephine C. Adams

**Affiliations:** ^1^Heart, Lung, Blood and Vascular Medicine Institute, Division of Pulmonary, Allergy and Critical Care Medicine, Department of Medicine, University of Pittsburgh, Pittsburgh, Pennsylvania; and; ^2^School of Biochemistry, University of Bristol, Bristol, United Kingdom

central to the homeostatic life of cells is the need to coordinate responses to external and internal changes. These processes become even more important in the context of the sustained cell-cell interactions that take place in multicellular organisms. As amply studied in metazoans, intricate mechanisms allow communication between the cell of production (autocrine), as well as similar and dissimilar cells both locally (paracrine) and over great distances (endocrine). These mechanisms of cell communication have been categorized into families of signal transducers, and this knowledge has provided an intellectual framework within which to understand the molecular details of the processes. The classic examples of cell-to-cell communication relate to proteins, especially those secreted into the extracellular space. In general, such proteins interact with specific cell membrane receptors to alter membrane protein/domain organization and/or cytoplasmic/organelle and nuclear events and thus cell response. However, communication by secreted proteins is itself limited through the process of diffusion, which for large macromolecules may not be insignificant (10).

Over the last quarter century plus, demonstrations of signaling from outside to inside cells via protein ligands and receptors have paralleled technical advances in cell culture, protein biochemistry, and antibody development. In contrast, other less cumbersome, more diffusible moieties such as nitric oxide (NO), carbon monoxide (CO), and hydrogen sulfide (H_2_S) have been known to engage with proteins for some time. As early as 1891, H_2_S was reported to interact with hemoglobin (2), and by 1925 this interaction was confirmed for NO (1). However, the meaning of these interactions in terms of human health was not apparent until much later, although the deadly consequences of exposure to CO were known as early as the start of the 20th century (7). As small and highly mobile molecules, NO, CO, H_2_S (4), and other recent possible candidates such as ammonia (NH_3_), methane (CH_4_) and even hydrogen (H^+^) or hydroxyl (OH^−^), have been classified as gaso-transmitters. As a group, these agents share properties including being gases under physiologically relevant conditions, crossing cell membranes rapidly, being produced biochemically by proteins (excepting hydroxyl radical), and displaying discrete threshold levels of signaling (6). This shift in perspective from toxic agent or pollutant to active and important signaling molecules has promoted an abundance of research. Translational studies have defined roles for these agents in human health and disease, and this has resulted in clinical studies and in some instances new therapies. The first to reach the clinic as a therapeutic was NO, as an inhaled agent for newborn respiratory failure (1995, NCT00005776). Applications of NO to human disease were initially via surrogates that intersect the NO signaling cascade such as nitroglycerine (3, 9), nitrite/nitrate (8), blockers of phosphodiesterase activity to increase NO's second messenger guanosine monophosphate (5), and most recently NO itself (ClinicalTrials.gov Identifier: NCT01089439, others). Although CO (Identifier: NCT01523548, others) and H_2_S (Identifier: NCT02899364, others) have been applied in a limited number of trials including phase 3 trials (for CO only), it remains to be seen if these and other biogases or their surrogates will become drugs.

Gaso-transmitter science is advancing rapidly as an area of research. In this issue, *American Journal of Physiology-Cell Physiology* begins a Theme of Reviews on Gaso-Transmitters. In the first of this series, Dr. Csaba Szabo (11) of the University of Texas Medical Branch provides a comprehensive review of H_2_S. We hope that the series of Reviews will expand awareness in the scientific and medical community of these fascinating molecules and may also stimulate increased cross-disciplinary research. We thank the authors for their kind contributions of expert Reviews for this Theme.

## GRANTS

J. S. Isenberg is supported by National Institutes of Health Grants R01 HL-108954, R01HL112914, and 1R21EB017184. Support was also provided to J. S. Isenberg by the Institute for Transfusion Medicine, the Hemophilia Center of Western Pennsylvania and the Heart, Lung, Blood and Vascular Medicine Institute of the University of Pittsburgh School of Medicine. Research in J. C. Adams's laboratory is supported by MRC K018043.

## DISCLOSURES

J. C. Adams has no conflicts of interest, financial or otherwise, to declare. J. S. Isenberg serves as chair of the scientific advisory boards of Tioma Therapeutics, Inc. (St. Louis, MO) and Radiation Control Technologies, Inc. (Jersey City, NJ).

## AUTHOR CONTRIBUTIONS

J.S.I. and J.C.A. drafted manuscript; J.S.I. and J.C.A. edited and revised manuscript; J.S.I. and J.C.A. approved final version of manuscript.
